# Nano-Enriched and Autonomous Sensing Framework for Dissolved Oxygen

**DOI:** 10.3390/s150820193

**Published:** 2015-08-14

**Authors:** Nader Shehata, Mohammed Azab, Ishac Kandas, Kathleen Meehan

**Affiliations:** 1 Department of Engineering Mathematics and Physics, Faculty of Engineering, Alexandria University, Alexandria 21544, Egypt; E-Mail: ishac@vt.edu; 2 Bradley Department of Electrical and Computer Engineering, Virginia Tech, Blacksburg, VA 24061, USA; 3 Center of Smart Nanotechnology and Photonics (CSNP), SmartCI research center, Alexandria University, Alexandria 21544, Egypt; E-Mail: mazab@vt.edu; 4 Informatics Research Institute, City of Scientific Research and Technological Applications, New Borg El-Arab City, Alexandria 21934, Egypt; 5 School of Engineering, University of Glasgow, Glasgow G128QQ, UK; E-Mail: Kathleen.Meehan@glasgow.ac.uk

**Keywords:** ceria nanoparticles, oxygen sensing, sensing framework, data collection

## Abstract

This paper investigates a nano-enhanced wireless sensing framework for dissolved oxygen (DO). The system integrates a nanosensor that employs cerium oxide (ceria) nanoparticles to monitor the concentration of DO in aqueous media via optical fluorescence quenching. We propose a comprehensive sensing framework with the nanosensor equipped with a digital interface where the sensor output is digitized and dispatched wirelessly to a trustworthy data collection and analysis framework for consolidation and information extraction. The proposed system collects and processes the sensor readings to provide clear indications about the current or the anticipated dissolved oxygen levels in the aqueous media.

## 1. Introduction

Dissolved oxygen (DO) sensing in aqueous media is important for wide variety of applications including biomedical research, environmental monitoring and process control [1,2,3]. Among various approaches in current literature, fluorescence-based oxygen sensing technique has significant advantages over the electrochemical-based one, due to little oxygen consumption, there is no need for any reference electrode, and they are immune to exterior electromagnetic field interference [4,5,6,7,8,9]. One of the most promising optical nanostructures is ceria nanoparticles due to its oxygen capability storage, adequate sensitivity and low-cost synthesis [10,11]. This paper offers a comprehensive monitoring framework as an integration between nanotechnology and trustworthy wireless sensor networks.

The main objective of our work is to develop a complete sensing platform for real time monitoring of the DO concentration in aqueous media as part of an effort to monitor water quality [12,13]. Our system goes behind local, single location monitoring to a networked sensing of DO concentration at multiple locations, across streams, water treatment facilities, hydroponic farms, and aquafarms. The system receives the digitalized signal from one or more nanosensor(s), merge the collected data with the geographical location of the sensing element(s), and analyzes it using remote offsite management servers in a real-time fashion. 

As shown in the results, the main advantage of having such level of autonomic control and management is the ability to predict some of the sensor feedback based on the analysis of the overall network feedback [14,15,16]. Such ability facilitates optimizing the sensor power usage by controlling the activation periods leading to much better sensor utilization expanding the lifetime of the entire network and reducing the system cost. Further, such ability facilitates detecting; Fixing/excluding any misbehaving or problematic sensing nodes that can massively reduce the system accuracy. Additionally, the automated system could definitely overcome the problems of manual data collection that requires extensive effort and time. This capability enables almost continuous real-time monitoring of DO with means to identify and address sensor drift helping researchers to construct test models based on the flow of nutrients, pollutants, and other constituents in the monitored water supply.

## 2. Experimental Section 

Ceria nanoparticles are prepared using a chemical precipitation technique [17,18,19]. Initially, 0.5 g of cerium (III) chloride (heptahydrate, 99.9%, Aldrich chemicals) is added to 40 mL de-ionized water as a solvent. The solution is stirred at rate of 500 rpm for 24 h through two stages. In the first step, the solution heated to 50 °C in normal atmosphere while stirring; 1.6 mL of ammonia is then added to ensure that the solution becomes homogenous for 1.5 h at 50 °C. Then, the solution is stirred for 22.5 h at room temperature. The synthesized ceria nanoparticles are characterized using UV-Vis spectroscopy (dual beam PG 90+) to detect the absorbance dispersion, which is the logarithmic ratio between the light intensities in the absence and the presence of the material along the light path over a wide range of the optical spectrum. The absorbance dispersion were detected for the solutions containing the synthesized undoped and doped ceria nanoparticles in de-ionized water. The reference sample is de-ionized water. The spectrum range is detected from 300 nm to 800 nm. The synthesized nanoparticles are characterized using Perkin XRD, and imaged using Phillips TEM (EM420).

**Figure 1:**
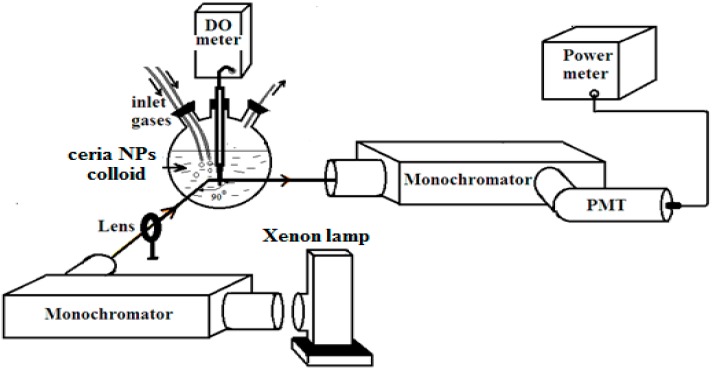
Experimental setup of optical sensing for dissolved oxygen.

The experimental apparatus used to correlate the quenching of the fluorescence from the colloidal ceria NPs with dissolved oxygen concentration in the aqueous solution is shown in Figure 1. The fluorescence spectroscopy system consists of Xenon lamp (Oriel instrument) followed by a monochromator, (a ¼ m Newport Cornerstone 260) with allowed light centered at 340 nm. The optical output from the monochromator is focused onto a three-neck flask containing distilled water solution of the synthesized ceria nanoparticles. Oxygen and nitrogen gases are fed through individual lines through a double-holes cork placed into one of the necks on the flask and controlled by a mass flow rate controller (MKS 247-C). The probe of a commercial DO meter (Milwaukee MW600 with a measurement range up to 19.9 mg/L) is inserted in the second neck of the flask to measure DO concentration. The fluorescence signal is collected from the colloidal solution scanned by a second Newport Cornerstone 260 monochromator, positioned at a 90^o^ angle to the first monochromator for minimum scattering effects. Then, a photomultiplier tube (Newport PMT 77340) is connected to a power meter (Newport Power meter 2935C) for fluorescence intensity monitoring.

**Figure 2:**
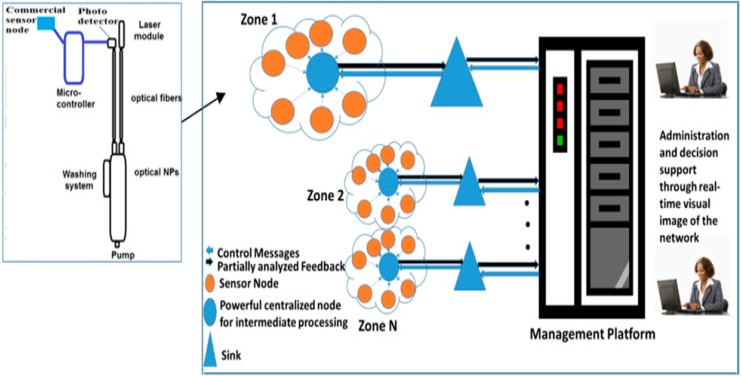
The sensing framework.

Regarding autonomous sensor management, Figure 2 is a block diagram of the sensor framework where the main sensing element is interfaced with an autonomously managed wireless sensor network for water quality monitoring. The sensing function will be interconnected to the cyber layer for control, management, and monitoring. 

The nanosensor is interfaced an A2D chip, a powerful microcontroller chip, a GPS chip, and wireless transmission module. The microcontroller is programed to control the activation and deactivation of the sensing element, and control the measurement configuration if necessary. The microcontroller receives its guideline form a remote management server. Each sensor has a unique identifier that is used in all transmissions along with the geographical location of the sensor in case of mobility. The system is built to be as generic as possible, allowing more sensing elements to be attached to the same sensing elements if the application requires that.

The system is built to scale; sensors are grouped into different zones. The sensor feedback and location is dispatched frequently either upon event change, query, or based on a predetermined schedule to a central data collection node at each zone. This node applies partial analysis and data grouping and dispatch a comprehensive zone status-report to the management server for further analysis and guidance. The algorithms used to establish such analysis is application dependent. We devised a simple case study with a simple model for the excremental study just to reflect the effect of such automation on the quality of the system output.

## 3. Results and Discussion

### 3.1. Optical Nanoparticles Characterization

**Figure 3:**
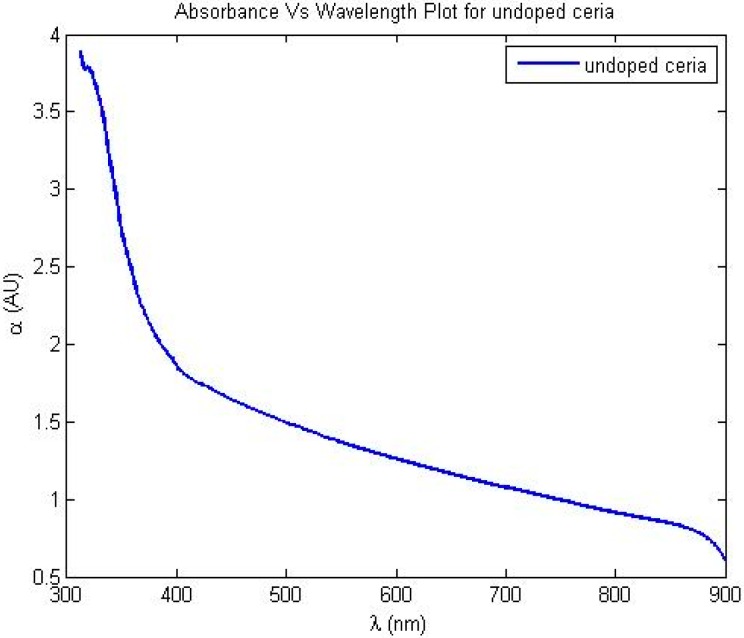
Absorbance dispersion for the synthesized ceria nanoparticles.

Figure 3 shows the resulted absorbance dispersion of the synthesized nanoparticles. Based on the resulted absorbance measurements, the allowed direct bandgap semiconductor of the synthesized nanoparticles can be found through the following equation [20]

**Figure 4:**
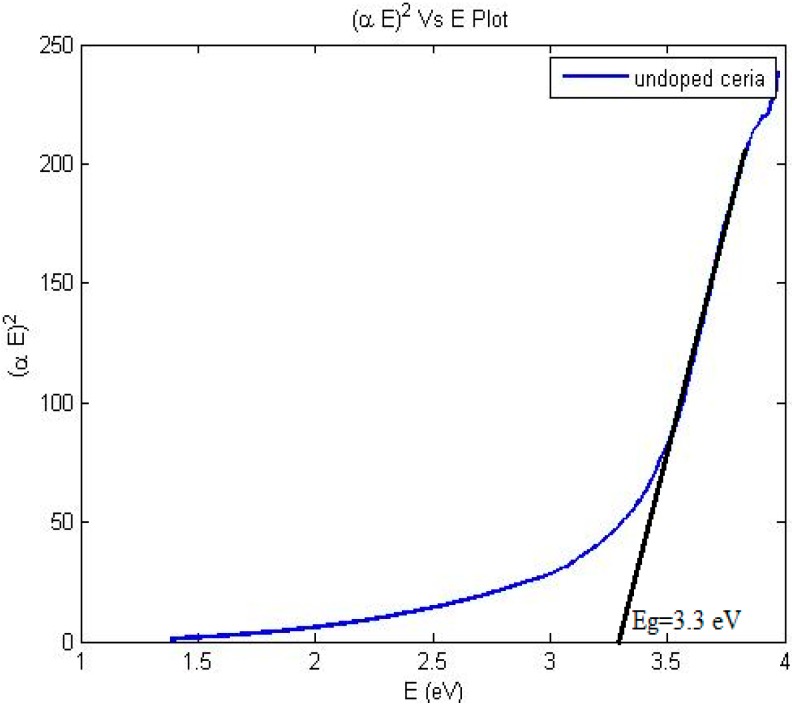
Bandgap calculations of the synthesized ceria nanoparticles.

**Figure 5:**
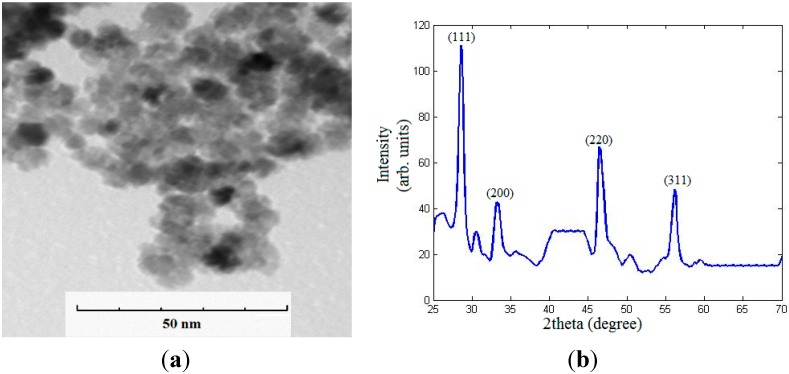
TEM image and XRD pattern of the synthesized ceria nanoparticles. (**a**) TEM image; (**b**) XRD pattern.

(1) αhv=A*(hv−Eg)12

where A* is a constant for the given material depending on its refractive index and effective masses of both electrons and holes, *h* is Planck’s constant, v is the absorbed frequency, and $E_{g}$ is the direct allowed bandgap energy. Then, (αhv)^2^ is plotted with photon energy, and the intersection with x-axis gives the value of bandgap energy. The calculated $E_{g}$ is found as shown in Figure 4. Figure 5 shows transmission electron microscope (TEM) image and X-ray diffraction (XRD) analysis of the synthesized nanoparticles with mean diameter around 6 nm.

### 3.2. DO Sensing

**Figure 6:**
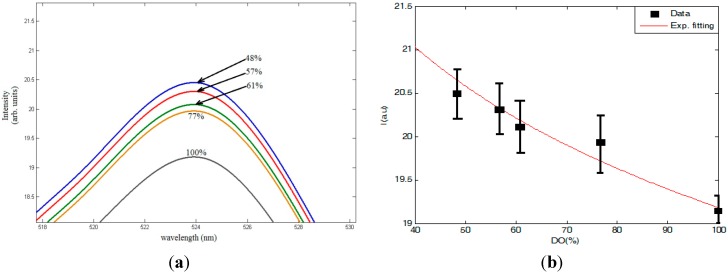
(**a**) Visible flouresence spectra at different DO concentration; and (**b**) Fluorescence peak intensity variation with fitting.

**Figure 7:**
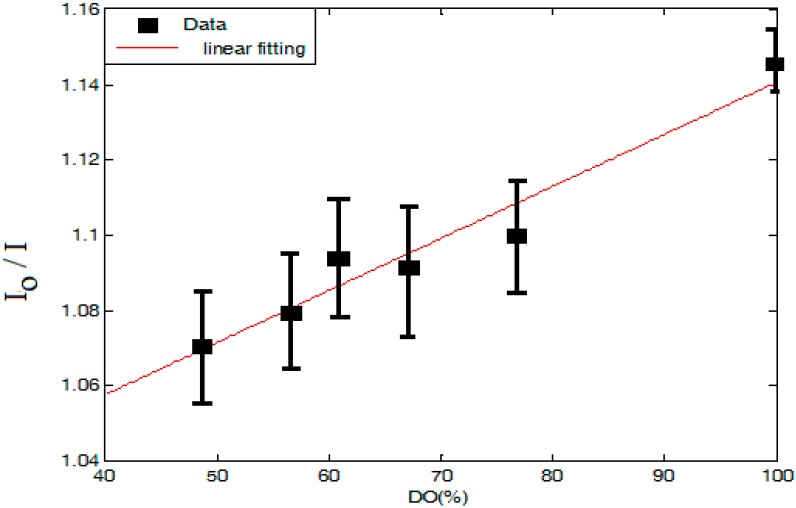
Relative peak intensity change with DO variation.

Figure 6 shows the change of the visible fluorescence emission intensity at 520 nm from the ceria nanoparticles with increasing DO concentration, under near-UV excitation. The relative intensity compared to the peak fluorescence intensity from the ceria nanoparticles at zero DO is shown in Figure 7. The value of *I_o_* could not be found experimentally as the DO concentration never reached zero even when there was no inlet flow of oxygen or nitrogen. We speculate that this is due to a release of oxygen stored in the ceria lattice when the nanoparticles are introduced into the solution. Therefore, *I_o_* is calculated by forcing the linear fit of the data to include a point for *I_o_/I* = 1 when DO = 0 mg/L. Regarding the error bars shown on both figures, during the detection of the emitted fluorescence at each stabilized DO concentration, the second monochromator is adjusted at the wavelength of peak intensity; ~520 nm. Then, the power meter records the maximum amplitude for 5 s. Hence, the mean value of the maximum amplitudes obtained during this time period is calculated and the error bars represent the minimum and maximum amplitudes of the peak fluorescence intensity around the mean value.

### 3.3. Detection Effectiveness

Regarding the experimental case study, our primary goal is to build a smart sensing framework where participating nodes cooperate to reflect a real-time image of the DO concentration on a large-scale acoustic media [21,22]. The main goal from this simulation is to determine the optimal configuration for the network operating in a remote location to guarantee efficient operation. The experiments were conducted with two densities (Sensors/Network) settings, Low/High. As we are operating in a remote and untrustworthy location, we intentionally impeded some malicious nodes that work on interrupting the system operation. We used two radio range settings, medium and high. Results showed the effect of increasing the density (cooperation) on the data accuracy, and the effect of increasing the maliciousness effect on the signal accuracy, and energy consumption for each case. Finally, we also tested the effect of extending the communication range on the energy consumption and the accuracy for the two densities. Table 1 shows the simulation parameters used for performance evaluation analysis.

**Table 1:** Simulation parameters.

Density	# Sensors	# Clients	# Malicious Nodes	# Servers	Radio Range
Low	30	15	3~9	6~12	6~12
High	200	100	20~80	80~120	6~12~24

**Figure 8:**
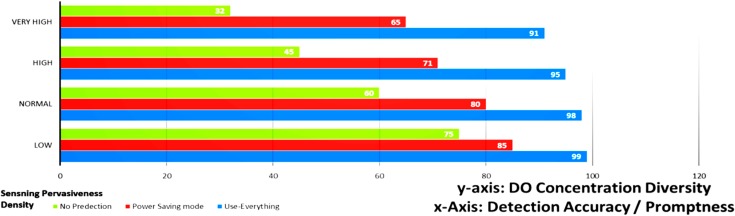
Detection effectiveness (accuracy/ promptness).

Figure 8 presents a simple performance evaluation of the proposed sensor network in a simulated scenario to illustrate the value of a fully integrated sensor network with the sensing framework with respect to the accuracy of the calculated DO concentration and response time of the system as measures of effectiveness. The automated data collection and analysis of the data from the nanosensor network with the DO prediction mechanism exhibits significant improvements in DO detection accuracy and promptness over the two other methods. The experiment tested four different sensor densities (number of sensors/meter) to test the system ability to scale. At each case, we evaluated the scenario of using smart data collection with prediction of DO concentration verses two modes, power saving (regular power efficient detect and dispatch) and extreme power usage (use all nodes all time).

With no prediction, and low power usage, the active number of nodes were not that high, and the accuracy were not as good as with the prediction and power saving. With prediction on, the management framework integrates other sources of information like the flow rate and direction of the media predicting some of the readings of sensors on the path. Such predictions minimize the activation time of these sensors and save a large some of energy. Finally, the use everything mode, this mode can be used on emergency cases. In this mode, we use all the sensors to monitor closely the DO levels. That mode consumes too much energy and massively reduces the sensor network lifetime.

**Figure 9:**
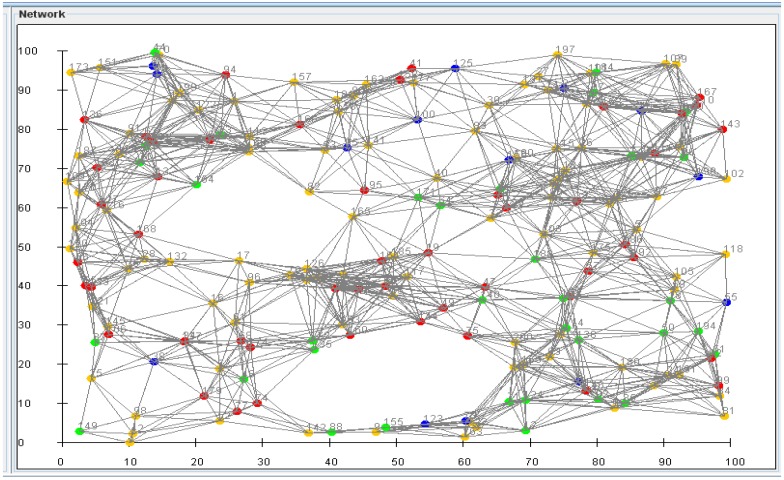
The interconnected sensor network.

**Figure 10:**
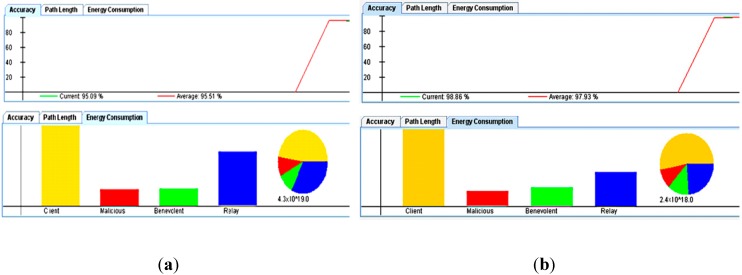
Study of the effect of maliciouesness: (**a**) High maliciousness; and (**b**) Low maliciousness.

Figure 9 and Figure 10 reflects the results of using only high density node scenario. Figure 9 shows the effect of increasing the malcisousness on the accurecy and power consumbtion. The effect of maliciousness on power consumption and accuracy is obvious, however, due to the automatic exclusion of such nodes from the network by our smart reputation manager, the maliciousness effect on the system accuracy is insignificant (~3%). The minor effect is mainly due to the small period that these malicious nodes were part of the network before being detected by the management platform.

**Figure 11:**
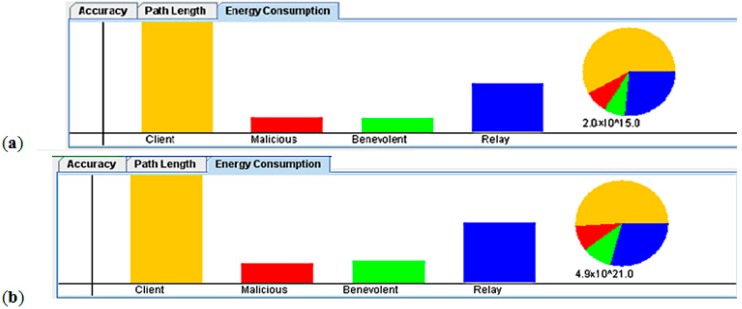
The effect of increaseing the radio range: (**a**) Low radio range; and (**b**) High radio range.

Figure 11 shows the clear effect of increasing the radio range on the power consumption. The use of high-energy transmission uses too much power, shortening the sensor node lifetime. Therefore, the effect of using prediction in minimizing the activation of such sensors can massively increase the sensor network lifetime. The main advantage of having such automated control that is fully aware of the system operational settings is the ability to adapt to the various operational-environment changes while maintaining high level of accuracy and maximizing the sensor network lifetime.

## 4. Conclusions

This work presents the integration between physical sensing of dissolved oxygen (DO) using the fluorescence quenching of ceria nanoparticles and the trustworthy data collection analysis. Our analysis shows clear change in the visible fluorescence intensity with the variation of the DO ratio. The reason is the developed oxygen vacancies concentration formed inside ceria nanoparticles, which act as DO receptor. Additionally, results showed the clear effect of the trustworthy automated data collection and smart analysis platform on the effectiveness, efficiency, and quality of detection. Having such system is essential for easy data consolidation and information extraction. Different experiments were conducted to show the effect of having such automation in enhancing the sensor and the network lifetime, even in the presence of malicious nodes. The proposed system presents an optimum solution for a comprehensive automated management of DO sensors in the aqueous media. Also, the automation platform is built to be generic and can be easily modified to be used with wide variety of other applications and sensing elements achieving wide scope of applications.

## References

[1] Warburton P.R., Sawtell R.S., Watson A., Wang A.Q. (2001). Failure prediction for a galvanic oxygen sensor. Sens. Actuators B Chem..

[2] Acosta M.A., Ymele-Leki P., Kostov Y.V., Leach J.B. (2009). Fluorescent microparticles for sensing cell microenvironment oxygen levels within 3D scaffolds. Biomaterials.

[3] Mohyeldin A., Garzón-Muvdi T., Quiñones-Hinojosa A. (2010). Oxygen in stem cell Biology: A critical component of the stem cell niche. Cell Stem Cell.

[4] Chu C.S., Lo Y.L. (2010). Optical fiber dissolved oxygen sensor based on Pt(II) complex and core-shell silica nanoparticles incorporated with sol–gel matrix. Sens. Actuators B Chem..

[5] Maskell W.C. (1987). Inorganic solid state chemically sensitive devices: electrochemical oxygen gas sensors. J. Phys. E Sci. Instrum..

[6] Sanghavi R., Nandasiri M., Kuchibhatla S., Jiang W., Varga T., Nachimuthu P., Engelhard M.H., Shutthanandan V., Thevuthasan S., Kayani A. (2011). Thickness dependency of thin-film samaria-doped ceria for oxygen sensing. IEEE Sens. J..

[7] Wang X., Wolfbeis O.S. (2014). Optical methods for sensing and imaging oxygen: materials, spectroscopies and applications. Chem. Soc. Rev..

[8] Chen L., Xu S., Li J. (2011). Recent advances in molecular imprinting technology: current status, challenges and highlighted applications. Chem. Soc. Rev..

[9] Mistlberger G., Klimant I. (2010). Luminescent magnetic particles: structures, syntheses, multimodal imaging, and analytical applications. Bioanal. Rev..

[10] Shehata N., Meehan K., Leber D. (2012). Fluorescence quenching in ceria nanoparticles: dissolved oxygen molecular probe with relatively temperature insensitive Stern-Volmer constant up to 50^o^C. J. Nanophotonics.

[11] Shehata N., Meehan K., Hudait M., Jain N., Gaballah S. (2014). Study of optical and structural characteristics of ceria nanoparticles doped with negative and positive association lanthanide elements. J. Nanomater..

[12] Ramamoorthy R., Dutta P.K., Akbar S.A. (2003). Oxygen sensors: Materials, methods, designs and applications. J. Mater. Sci..

[13] Oczkowski A., Nixon S. (2008). Increasing nutrient concentrations and the rise and fall of a coastal fishery: A review of data from the Nile Delta. Estuar. Coast. Shelf Sci..

[14] Azab M., Eltoweissy M. Bio-inspired evolutionary sensory system for cyber-physical system defense. Proceedings of the 2012 IEEE Conference on Technologies for Homeland Security (HST).

[15] Hill C., Sippel K. Modern Deformation Monitoring: A Multi Sensor Approach. http://citeseerx.ist.psu.edu/viewdoc/summary?doi=10.1.1.127.9526.

[16] Garich E.A. (2007). Wireless Automated Monitoring For Potential Landslide Hazards. Master’s Thesis.

[17] Chen H., Chang H. (2004). Homogeneous precipitation of cerium dioxide nanoparticles in alcohol/water mixed solvents. Coll. Surf. A.

[18] Shehata N., Meehan K., Hassounah I., Hudait M., Jain N., Clavel M., Elhelw S., Madi N. (2014). Reduced erbium-doped ceria nanoparticles: One nano-host applicable for simultaneous optical down- and up-conversions. Nanoscale Res. Lett..

[19] Shehata N., Meehan K., Leber D. (2013). Study of fluorescence quenching in aluminum-doped ceria nanoparticles: Potential molecular probe for dissolved oxygen. J. Fluoresc..

[20] Pankove J. (1971). Optical Processes in Semiconductors.

[21] Kartakis S., Abraham E., McCann J. (2015). WaterBox: A Testbed for Monitoring and Controlling Smart Water Networks. CySWater’15.

[22] Fattoruso G., Tebano C., Agresta A., Lanza B., Antonio B., Vito S.D., Francia G.D. (2015). A SWE architecture for real time water quality monitoring capabilities within smart drinking water and wastewater network solutions. Comput. Sci. Appl..

